# Non-linear impact of glutathione depletion on *C. elegans* life span and stress resistance

**DOI:** 10.1016/j.redox.2016.12.003

**Published:** 2016-12-06

**Authors:** Nadine Urban, Dimitrios Tsitsipatis, Franziska Hausig, Katrin Kreuzer, Katrin Erler, Vanessa Stein, Michael Ristow, Holger Steinbrenner, Lars-Oliver Klotz

**Affiliations:** a Institute of Nutrition, Department of Nutrigenomics, Friedrich-Schiller-Universität Jena, D-07743 Jena, Germany; b ETH Zurich (Swiss Federal Institute of Technology), Schorenstrasse 16, Building SLA C7, CH-8603 Schwerzenbach-Zurich, Switzerland

**Keywords:** BCA, bicinchoninic acid, *C. elegans*, *Caenorhabditis elegans*, CTL, catalase, DAF-16, abnormal dauer formation 16, DEM, diethyl maleate, DMSO, dimethyl sulfoxide, DTNB, 5,5'-dithiobis-(2-nitrobenzoic acid), *E. coli*, *Escherichia coli*, EGL, egg laying defective, FoxO1, forkhead box class O 1, GCS, γ-glutamylcysteine synthetase, GFP, green fluorescent protein, GPX, glutathione peroxidase, GSH, glutathione, GSSG, glutathione disulfide, GST, glutathione S-transferase, ICL, isocitrate lyase, mBBr, monobromobimane, NGM, nematode growth medium, Nrf2, nuclear factor erythroid 2-related factor 2, PBST, phosphate-buffered saline with Tween 20, ROS, reactive oxygen species, SKN-1, skinhead 1, SOD, superoxide dismutase, TRX, thioredoxin, TRXR, thioredoxin reductase, Glutathione, *C. elegans*, Aging, Stress resistance, Thiol, γ-glutamylcysteine synthetase, Hormesis

## Abstract

The redox environment in cells and organisms is set by low-molecular mass and protein-bound thiols, with glutathione (GSH) representing a major intracellular redox buffer. Subtle thiol oxidation elicits signal transduction processes and adaptive responses to cope with stressors, whereas highly oxidizing conditions may provoke cell death. We here tested how thiol depletion affects life span, stress resistance and stress signaling in the model organism *Caenorhabditis elegans*. Diethyl maleate (DEM), an α,β-unsaturated carbonyl compound that conjugates to GSH and other thiols, decreased *C. elegans* life span at a concentration of 1 mM. In contrast, low and moderate doses of DEM (10–100 µM) increased mean and maximum life span and improved resistance against oxidative stress. DEM-induced life span extension was not detectable in worms deficient in either the FoxO orthologue, DAF-16, or the Nrf2 orthologue, SKN-1, pointing to a collaborative role of the two transcription factors in life span extension induced by thiol depletion. Cytoprotective target genes of DAF-16 and SKN-1 were upregulated after at least 3 days of exposure to 100 µM DEM, but not 1 mM DEM, whereas only 1 mM DEM caused upregulation of *egl-1*, a gene controlled by a p53-orthologue, CEP-1. In order to test whether depletion of GSH may elicit effects similar to DEM, we suppressed GSH biosynthesis in worms by attenuating γ-glutamylcysteine synthetase (*gcs-1*) expression through RNAi. The decline in GSH levels elicited by *gcs-1* knockdown starting at young adult stage did not impair viability, but increased both stress resistance and life expectancy of the worms. In contrast, *gcs-1* knockdown commencing right after hatching impaired nematode stress resistance and rendered young adult worms prone to vulval ruptures during egg-laying. Thus, modest decrease in GSH levels in young adult worms may promote stress resistance and life span, whereas depletion of GSH is detrimental to freshly hatched and developing worms.

## Highlights

• Modest GSH depletion through knockdown of *gcs-1* elevates nematodal life span.
• Moderate thiol depletion by DEM elevates stress resistance and life span of nematodes.
• Life span extension by DEM is mediated through transcription factors DAF-16 and SKN-1.
• A high DEM dose is toxic for nematodes despite nuclear translocation of DAF-16.

## 1 Introduction

Glutathione (γ-L-glutamyl-L-cysteinylglycine; GSH) is considered the major intracellular low-molecular-mass thiol in eukaryotes, present in millimolar concentrations in cells. GSH has a pivotal role in antioxidant defense, serving as cosubstrate for glutathione peroxidase (GPX)-catalyzed reductions of H_2_O_2_ and lipid hydroperoxides. GPX-mediated removal of hydroperoxides is accompanied by oxidation of GSH to glutathione disulfide (GSSG), whose reduction back to GSH is catalyzed by glutathione reductase and occurs at the expense of NADPH [1].

Under conditions of oxidative stress, GSH also forms mixed disulfides with cysteinyl residues in proteins. This reversible S-glutathiolation may protect proteins against cysteine oxidation beyond the disulfide stage, thereby preventing irreversible protein inactivation and degradation during stress [2]. In addition, GSH forms conjugates with xenobiotic and endogenous electrophilic compounds as part of phase II xenobiotic metabolism and thus assists in detoxification and excretion of said compounds [3].

Therefore, GSH serves as a major cellular line of defense against oxidative stimuli at several levels. Accordingly, depletion of cellular GSH may be detrimental to cells, and an elevated rate of GSH biosynthesis to counteract such depletion may occur as part of an adaptive response of cells to (oxidative) stress. For example, an adaptation at the level of GSH biosynthesis was demonstrated in mammalian cells in response to such stimuli as acrolein or cumene hydroperoxide and was suggested to occur through nuclear factor-erythroid 2-related factor 2 (Nrf2)-dependent signaling [4] (for a recent review on Nrf2, see [5]).

This dichotomy between GSH depletion as being detrimental but stimulating GSH *de novo* synthesis bears the question whether there is a difference between strong and minor GSH depletion with respect to biological outcome; in other words: is there an extent of GSH depletion that will enhance cellular stress resistance rather than promote cell death?

In order to investigate the role of GSH depletion in the regulation of stress resistance at an organismal level and to test for any potential consequences for organismal life span, we here employ the nematode *Caenorhabditis elegans* (*C. elegans*) as an animal model. *C. elegans* is widely used in mechanistic studies on aging, toxicity and metabolism owing to its relatively short lifespan as well as the availability of well-established protocols for its maintenance, treatment and genetic manipulation [6]. Moreover, these nematodes share a high degree of genetic, biochemical and physiological similarity with humans [6], including highly conserved pathways involved in the regulation of life span and stress resistance [7].

Herein, we investigate the effects of glutathione depletion in *C. elegans* through pharmacological (using thiol-modulating agents) or genetic (using RNA interference) approaches, with respect to stress signaling, stress resistance and consequences for lifespan. We demonstrate that, depending on developmental stage of the nematode, glutathione depletion may be either beneficial or aggravate the impact of stressful stimuli.

## 2 Materials and methods

### 2.1 Materials

*C. elegans* strains were provided by the Caenorhabditis Genetics Center (CGC, University of Minnesota, USA), which is supported by the National Institutes of Health-Office of Research Infrastructure Programs: wild-type Bristol N2; EU31 [skn-1(zu135)]; CF1038 [daf-16(mu86)]; TJ356 zIs356 [daf-16p::daf-16a/b::GFP+rol-6]. *E. coli* strains OP50 and OP50i were also received from CGC. For RNAi knockdown experiments *E.coli* HT115 *gcs-1* clones were derived from an Ahringer library (Source BioScience, Nottingham, UK; [8]).

Chemicals were purchased from Sigma-Aldrich (Munich, Germany) and Carl Roth (Karlsruhe, Germany) unless stated otherwise. Primers were obtained from Life Technologies (Darmstadt, Germany).

### 2.2 C. elegans maintenance and treatment

Nematodes were grown, maintained and treated at 20 °C on nematode growth medium (NGM) agar plates spotted with *E. coli* OP50 as food source, as described elsewhere [9]. For stress resistance assays, heat-inactivated bacteria (45 min at 65 °C) were used.

Stock solutions of diethyl maleate (DEM), menadione and diamide were prepared in DMSO. The compounds or the solvent control (0.1% DMSO) were added directly to the agar during preparation of plates. Treatment with the compounds started 64 h after synchronization of the nematodes, unless stated otherwise. Synchronization was performed by washing, followed by centrifugation to separate the eggs from the nematodes. Eggs were transferred to fresh NGM agar plates and allowed to hatch and grow for 64 h before being transferred to incubation plates containing the respective compounds. For long-term incubations, nematodes were washed off the plates with S-basal medium on a daily basis, and were transferred to freshly prepared NGM agar plates to separate nematodes from progeny.

For RNAi experiments, 1 mM isopropyl-β-D-thiogalactoside (IPTG), 100 µg/ml ampicillin and, if necessary, 12.5 μg/ml tetracycline were added to NGM agar. Agar plates were spotted with *E. coli* HT115 containing L4440 empty vector or *gcs-1* cDNA fragment in L4440 on the evening before and allowed to dry overnight. Incubations with RNAi bacteria started either immediately or 64 h after synchronization of nematodes.

### 2.3 Life span assays

Life span analyses were conducted at 20 °C. 64 h after synchronization, nematodes were manually transferred to fresh NGM agar plates containing the respective compound or solvent control. For the first 10 days, nematodes were transferred daily to avoid overcrowding and for separation of adult nematodes from their offspring. After the reproduction period, worms were transferred every second day. On day 12, nematodes were transferred to NGM agar plates containing 200 μg/ml streptomycin and covered with the streptomycin-resistant *E. coli* strain OP50i to avoid contamination. Worms showing no movement, no reaction to gentle stimulation and no pharyngeal pumping were scored as dead. Worms lost or disintegrated due to internal hatchings were censored. Experiments were performed in quintuplicates and at least two independent times.

Life span assays with *gcs-1*-specific RNA interference were conducted as follows. Immediately after (experiments in Fig. 8), or 64 h after synchronization (see Fig. 6) nematodes were transferred to NGM agar plates containing 1 mM IPTG, 100 µg/ml ampicillin and, if necessary, 12.5 μg/ml tetracycline and spotted with *E. coli* HT115 containing empty vector L4440 or vector containing a *gcs-1* cDNA fragment (Ahringer library [8]). For the first 10 days, nematodes were transferred to fresh plates daily; thereafter, they were transferred every other day. Experiments were performed in quintuplicates.

### 2.4 Stress resistance assays

64 h after synchronization, N2 wild-type nematodes were incubated for 5 days on agar plates supplemented with 100 μg/ml ampicillin, the respective compounds or solvent controls, and spotted with heat-inactivated *E. coli* OP50. Subsequently, N2 wild-type nematodes were transferred to NGM agar plates containing 10 mM of the superoxide-generating compound paraquat (Acros Organics, Geel, Belgium) and spotted with heat-inactivated *E. coli* OP50. Stress resistance assays were conducted as triplicates and repeated at least once. Surviving worms were counted every day as described for life span assays. Worms that crawled off the plates or disintegrated due to internal hatchings were censored.

Stress resistance assays with *gcs-1* RNA interference were performed as follows: either right after synchronization, or 64 h after synchronization (L4), nematodes were transferred to plates containing 1 mM IPTG, 100 µg/ml ampicillin and spotted with *E. coli* HT115 containing empty vector L4440, or L4440 containing a *gcs-1* cDNA fragment. Subsequently, nematodes were incubated with RNAi bacteria until 5 days after L4 stage. Afterwards, nematodes of each group were transferred to plates containing 300 mM paraquat. Survival of worms was counted every hour.

### 2.5 Analysis of subcellular localization of DAF-16::GFP in C. elegans

24 h after synchronization, nematodes of the transgenic strain TJ356 stably expressing a DAF-16::GFP fusion protein [10] were transferred to NGM agar plates containing the respective compound or solvent control for additional 24 h. Subsequently, around 40 L3 larvae of each group were placed on microscope slides coated with 3% agarose, anaesthetized with 10 mM sodium azide, and covered with coverslips. Cellular localization of DAF-16 was analyzed by fluorescence microscopy on an Axio Observer D1 fluorescence microscope (Zeiss, Göttingen, Germany) using appropriate filters (ex. 472±30 nm, em. 520±35 nm). The nematodes were grouped into three categories (“nuclear”, “cytosolic” or “cytosolic/nuclear”) according to the predominant localization of the DAF-16::GFP fusion protein. The experiment was performed at least three independent times. For analysis of subcellular localization of DAF-16::GFP following *gcs-1* RNA interference, nematodes (*C. elegans* TJ356) were transferred to plates spotted with *E. coli* HT115 (containing L4440 empty vector or containing a *gcs-1* cDNA fragment for RNAi) 24 h after synchronization. They were held on these plates for 24 h or 48 h. Subsequently, nematodes were scored with respect to the predominant subcellular localization of DAF-16. Three independent experiments were performed.

### 2.6 In vivo determination of thiols with monobromobimane (mBBr)

Intracellular thiol levels were assessed using the fluorogenic probe monobromobimane (mBBr) which forms a fluorescent thiol-bimane adduct. 64 h after synchronization, wild-type nematodes were collected from plates, resuspended in S-basal, centrifuged and pellets distributed to wells of a 12-well plate (Sarstedt, Nümbrecht, Germany) containing 2 ml PBST/well, supplemented with the respective thiol modulating compound or vehicle control. Following an exposure time of 2 h at 20 °C, worms were washed twice with S-basal and transferred to NGM agar plates spotted with a mixture of 500 µL heat-inactivated *E. coli* OP50 and 100 µL of a 1 mM mBBr stock solution (in DMSO). They were incubated on these plates for an additional 2 h. Subsequently, worms were washed off the plates and transferred to fresh NGM agar plates spotted with *E. coli* OP50 for 1 h in order to remove residual dye from the gut. Worms were then washed off the plates, resuspended with S-basal and transferred to a 96-well plate (FLUOTRAC™, Greiner Bio-One, Frickenhausen, Germany). Fluorescence was measured in a microplate reader (CLARIOstar, BMG Labtech, Offenburg, Germany) using well-scanning mode (excitation: 360 nm; emission: 460 nm). To normalize the fluorescence signals, worms were removed from the wells, sonicated and centrifuged, and the obtained supernatant was used for protein quantitation (see below). The experiment was performed 5 independent times.

### 2.7 In vitro determination of thiols using DTNB

64 h after synchronization, worms were washed twice with S-basal and transferred to NGM agar plates containing the respective compound to be tested for its effects on thiols or the corresponding solvent control for 3 h. Worms were then washed off the plates, centrifuged and pellets shock-frozen in liquid nitrogen. Worms were lysed by grinding in liquid nitrogen and adding 250 µL of S-basal containing proteinase inhibitors. After sonication thiols in supernatants of lysates were assessed using 5,5′-dithiobis (2-nitrobenzoic acid) (DTNB) [11]. Absorbance of thionitrobenzoate released from DTNB upon interaction with thiols was measured at 412 nm, related to a GSH standard and normalized to protein content of lysates. Three independent experiments were performed. Protein content of worm lysates was assessed according to Bradford [12] or using bicinchoninic acid (BCA) according to manufacturers’ instructions (Bio-Rad Laboratories AG, Munich, Germany, and Thermo Scientific, Waltham, MA, USA, respectively). Absorbance was measured with a microplate reader (CLARIOstar, BMG Labtech, Offenburg, Germany).

### 2.8 Analysis of GSH levels in C. elegans

GSH in *C. elegans* was determined by HPLC (PU-1580, Jasco, Gross-Umstadt, Germany) after derivatization of thiols with orthophtaldialdehyde (OPA) and fluorometric detection (FP-920, Jasco) according to Lüersen *et al.* [13] and Neuschwander-Tetri & Roll [14]. Harvested worms were homogenized with mortar and pestle under liquid nitrogen, with 200 µL of cold S-basal containing protease inhibitors added. Homogenates were thawed on ice, sonicated and centrifuged to separate debris. After centrifugation, proteins were precipitated by addition of 25 µL of cold 2 N perchloric acid to 50 µL of the supernatant, followed by incubation on ice for 1 min. This mixture was neutralized by addition of 200 µL of 0.5 M sodium phosphate buffer (pH 7.0), followed by centrifugation for 10 min at 4 °C. 50 µL of the neutralized supernatant was used for derivatization with 50 µL of OPA [2% (w/v) in 0.1 M sodium borate, pH 9]. Separation was performed by gradient elution on a ZORBAX Bonus RP column (4,6×250 mm; Agilent) at a flow rate of 1 ml/min. Eluents were (A) 98% of 50 mM sodium acetate (pH 7) / 2% acetonitrile (VWR) and (B) 80% acetonitrile / 20% 50 mM sodium acetate (pH 7.0). Peaks were detected at 420 nm after excitation at 350 nm. GSH was normalized to protein content (determined as above) of the respective sample. At least three independent experiments were performed.

### 2.9 RNA extraction and quantitative reverse transcriptase-PCR (qRT-PCR)

64 h after synchronization, worms were distributed to NGM agar plates containing the desired concentration of DEM or solvent control (DMSO), or to plates spotted with *E. coli* HT115 (containing L4440 empty vector or containing a *gcs-1* cDNA fragment for RNAi). Worms were washed and transferred to new plates (also containing DEM or DMSO or spotted with *E. coli* HT115) daily, until the day of harvesting. Worms were collected at the respective time points and shock-frozen in liquid nitrogen. Total RNA was isolated using TRIzol reagent (Thermo Scientific). RNA (500 ng) was reversely transcribed using GoScript Reverse Transcriptase (Promega) or RevertAid Reverse Transcriptase (Thermo Scientific), according to the manufacturer's instructions, and subjected to qPCR analysis using SsoAdvanced Universal SYBR Green Supermix and a CFX Connect cycler (Bio-Rad Laboratories AG, Munich, Germany). *Act-1* was used as housekeeping gene for relative quantitation of mRNAs of interest. For confirmation of *gcs-1* knock-down, RNA was isolated from nematodes and reversely transcribed as described above. The cDNA was subjected to qPCR analysis, using *tba-1* as housekeeping gene. Sequences of PCR primers are compiled in Table 1.

### 2.10 Statistical analysis

Data are expressed as means+SEM unless stated otherwise. For lifespan and stress resistance assays, statistical calculations were performed using JMP software version 9.0 (SAS Institute Inc., Cary, NC, USA), applying the log-rank test. All other calculations were performed using GraphPad 5 (GraphPad Software, San Diego, California, USA). Statistical significances were calculated using Student's t-test (paired or unpaired, two-tailed), where appropriate. The minimum level of significance was set to p<0.05.

## 3 Results

### 3.1 Thiol depletion may enhance C. elegans lifespan

**Fig. 1:**
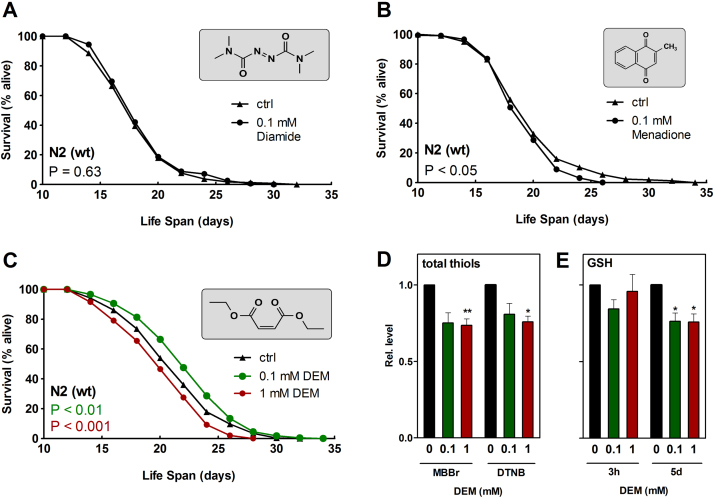
Thiol-depleting compounds modulate *C. elegans* life span. Survival rates of wild-type nematodes grown on (A) diamide (100 µM; P=0.63, log-rank test) (B) menadione (100 µM; P<0.0001, log-rank test) and (C) DEM at 100 µM (green, P<0.01, log-rank test) and 1 mM (red, P<0.001, log-rank test). Age-synchronized 64 h old wild-type nematodes were transferred to NGM agar plates supplemented with the respective compounds. Survival at 20 °C was monitored daily until the end of the reproduction period and every second day thereafter. Experiments were conducted in quintuplicates and were performed at least twice (for details, see Table 2). One representative survival curve is depicted. (D) Relative total thiol levels after 2 h of exposure to 100 µM DEM (green), 1 mM DEM (red) or 0.1% (v/v) DMSO as detected *in vivo* using monobromobimane (MBBr) or after 3 h of exposure to DEM measured *in vitro* with dithionitrobenzoate (DTNB). Thiol/protein ratios were calculated and normalized against the respective controls. Data are presented as means (MBBr, n=5; DTNB, n=3)±SEM. (E) Relative glutathione (GSH) levels in *C. elegans* after 3 h and 5 d of exposure to 100 µM (green) or 1 mM DEM (red). GSH/protein ratios were determined and normalized against the respective controls. Data are presented as means±SEM from 4 independent experiments. *p<0.05; ^**^p<0.01; Student´s t-test. (For interpretation of the references to color in this figure legend, the reader is referred to the web version of this article.)

Growth of *C. elegans* wild type (N2) worms in the presence of 100 µM of either of three different thiol-modulating agents had varying effects on life span. Diamide, a compound that directly oxidizes 2 GSH to GSSG [15]), did not affect life span (Fig. 1A). Menadione, a redox cycler and alkylating agent [16], [17], [18], caused a slight reduction in life span, in line with previously published reports [19] (Fig. 1B). In sharp contrast, growth in the presence of 100 µM diethyl maleate (DEM), an α,β-unsaturated carbonyl compound, significantly increased both mean and maximum life span of *C. elegans* (Fig. 1C). DEM forms adducts with thiols and has some selectivity for GSH by being a substrate for glutathione S-transferases [20]. In essence, it depletes GSH but does not necessarily cause its oxidation [21]. On the other hand, nematode life span was shortened upon exposure to a high concentration (1 mM) of DEM (Fig. 1C). We also assessed two lower DEM concentrations, 1 µM and 10 µM, both of which did not significantly affect *C. elegans* life span; Table 2 provides details on these life span experiments.

Interestingly, the analysis of total thiol levels employing two different approaches (either by *in vivo* analysis of thiol-dependent fluorescence using monobromobimane, or by detection of thiols in nematode lysates using Ellman's reagent, DTNB) indicated an approx. 25% decrease in nematode thiol content upon exposure of worms to 100 µM DEM (Fig. 1D) – despite the observed increase in life span. Analysis of GSH levels in worms exposed to DEM revealed that no more than a trend toward a decrease was achieved after 3 h of exposure, whereas significant GSH depletion by approx. 25% was seen after 5 days of cultivation in the presence of DEM (Fig. 1E). Absolute GSH concentrations determined in young adult *C. elegans* (controls; i.e. exposed to solvent controls or to control RNAi bacteria) was 9.7±5.3 nmol/mg protein (means±SD, n=28) and therefore close to previously reported values of approx. 12 nmol/mg [22] or approx. 40 nmol/mg [13]. The fact that overall thiols were depleted well before GSH in response to DEM suggests that protein thiols may be prone to modification by DEM prior to a decrease in levels of GSH. Somewhat surprisingly, no concentration dependence of changes in overall thiol (Fig. 1D) or GSH (Fig. 1E) levels was observed in the range between 0.1 and 1 mM DEM, indicating that neither cellular thiol nor GSH status are likely to be appropriate markers or determinants of the effects that thiol modulating agents might have on *C. elegans* viability.

Our further investigations focused (i) on the molecular mechanisms underlying the effects of DEM on *C. elegans* survival and stress resistance and (ii) on the role of GSH in modulating these effects.

### 3.2 Modulation of C. elegans stress resistance by DEM

**Fig. 2:**
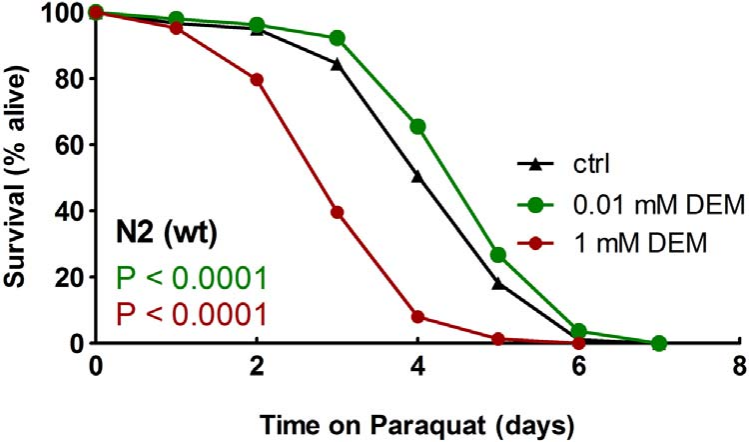
Survival of *C. elegans* on paraquat after exposure to DEM. Age-synchronized wild-type nematodes (64 h old) were incubated for 5 days on agar plates (containing 100 μg/ml ampicillin) supplemented with 10 µM DEM (green, P<0.0001, log-rank test), 1 mM DEM (red, P<0.0001, log-rank test) or 0.1% DMSO (ctrl, black) and spotted with heat-inactivated *E. coli* OP50. N2 wild-type nematodes were then transferred to NGM agar plates containing 10 mM paraquat and spotted with heat-inactivated bacteria. Survival of nematodes was determined. Stress resistance assays were conducted as triplicates and performed at least twice. For details, see Table 3. A representative experiment is depicted. (For interpretation of the references to color in this figure legend, the reader is referred to the web version of this article.)

Increases in nematode life span have been shown to be mechanistically linked to an enhanced capability of dealing with oxidative and other forms of stress, rendering stress resistance a determinant of longevity [23]. Therefore, we tested the impact of different DEM concentrations on the capability of *C. elegans* to survive oxidative damage induced by the redox cycler paraquat, which is known to generate intracellular superoxide [24]. Exposure to 10 µM DEM increased the mean and maximum survival of nematodes when held on agar containing a toxic dose of 10 mM paraquat (Fig. 2). On the other hand, 1 mM DEM impaired *C. elegans* survival in the presence of the redox cycler, resulting in diminished mean and maximum survival (Fig. 2). Different from the results obtained in life span analyses (Fig. 1C), 100 µM DEM had no effect on survival of paraquat-stressed worms (for details, see Table 3).

As one of the differences between life span analyses and stress assays was that worms were held on viable (life span) or heat-inactivated (stress assays) bacteria, we repeated the life span analysis under DEM treatment with heat-inactivated bacteria, in order to elucidate any potential interference of living *E.coli* OP50 bacteria with DEM acting on worms. Interestingly, a significant increase in mean and maximum life span of *C. elegans* was observed with 1 and 10 µM but not with 100 µM DEM under these conditions, whereas 1 mM DEM significantly shortened life span of the worms (Table 2).

In summary, DEM at low and non-toxic concentrations enhances both stress resistance and life span of *C. elegans*, and the absolute DEM concentrations required for the effect depend on whether viable or inactivated bacteria are employed for the respective assay.

### 3.3 Extension of C. elegans lifespan by low-dose DEM depends on transcription factors DAF-16 and SKN-1

DAF-16 and SKN-1, the *C. elegans* orthologues to mammalian FoxO and Nrf2 transcription factors, respectively, are major transcriptional regulators involved in the control of stress resistance and longevity [5], [25]. To elucidate their contribution to the modulation of life span by DEM, we employed mutant strains of *C. elegans* with inactivated DAF-16 or SKN-1 for further life span analyses.

**Fig. 3:**
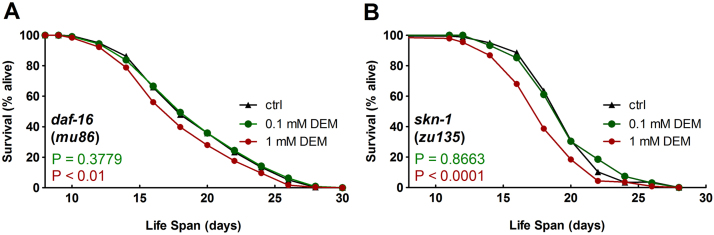
Life span analyses of *C. elegans daf-16* and *skn-1* mutant strains exposed to DEM. Age-synchronized nematodes (64 h old) were transferred to NGM agar plates containing DEM at 100 µM DEM (green), 1 mM DEM (red) or 0.1% DMSO (ctrl, black). Survival rates of (A) *daf-16* (mu86) k.o. nematodes and (B) *skn-1* (zu 135) k.o. nematodes at 20 °C were monitored daily until the end of the reproduction period and every second day thereafter. Experiments were conducted in quintuplicates and were performed at least twice (for details, see Table 4). P values were determined by log-rank test. Representative survival curves are depicted. (For interpretation of the references to color in this figure legend, the reader is referred to the web version of this article.)

The increase in life span of wild-type nematodes induced by moderate-dose (100 µM) DEM was not observed in a DAF-16-deficient mutant strain, whereas the absence of DAF-16 did not diminish the toxic (life-shortening) effect of high-dose (1 mM) DEM (Fig. 3**A**, details in Table 4). Similar results were also obtained in the SKN-1 mutant strain (Fig. 3**B**, Table 4). Thus, life span extension triggered by moderate-dose DEM depends on the action of both transcription factors, whereas the life-shortening effect of high-dose DEM was still observed in mutant strains and is therefore DAF-16 and SKN-1-independent.

### 3.4 Thiol depleting agents cause nuclear accumulation of DAF-16

**Fig. 4:**
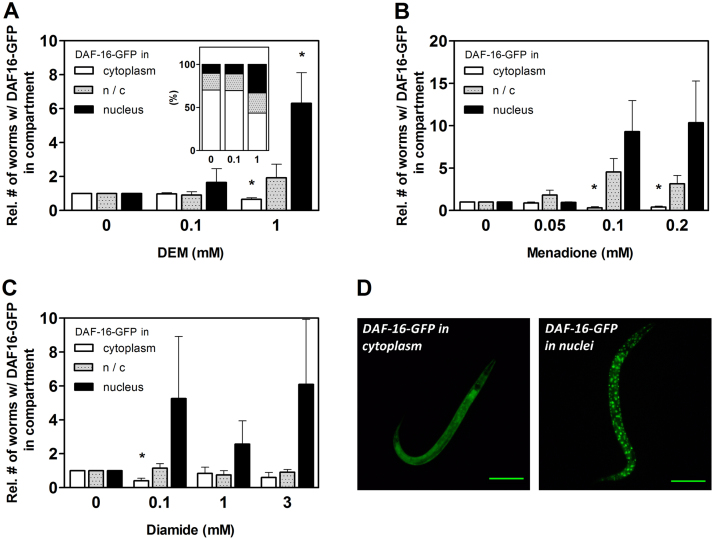
Subcellular localization of DAF-16::GFP in *C. elegans* exposed to thiol-modulating compounds. Age-synchronized L1 larvae of the *C. elegans* TJ356 strain stably expressing a DAF-16::GFP fusion protein were transferred to NGM agar plates supplemented with (A) DEM, (B) menadione or (C) diamide at the given concentrations. 0.1% DMSO was used as control (0 mM); exposure was for 24 h. For each independent experiment, nematodes of each treatment group were categorized with respect to the predominant localization of DAF-16::GFP fusion protein as detected under the fluorescence microscope. Data are means + SEM of the relative numbers of worms with predominantly cytoplasmic or nuclear DAF-16::GFP, or of worms with an intermediate phenotype (n/c) from at least 4 independent experiments. Data were normalized against respective control, which was set to 1. (A, inset) Distribution of DAF-16::GFP presented as fractions of 100%. *p<0.05, Student´s t-test. (D) Examples of worms with predominantly cytoplasmic (left) and nuclear localization (right) of DAF-16::GFP. Bar=100 µm.

A major consequence of stressful conditions such as oxidative stress, heat or UV irradiation is the accumulation of DAF-16 in the nucleus [26], usually resulting in its enhanced activity as transcription factor and up-regulation of genes involved in antioxidant protection. In order to test the impact of DEM on the intracellular localization of DAF-16, we employed a transgenic *C. elegans* strain stably expressing a DAF-16::GFP fusion protein [10]. Exposure of L1 stage worms to 100 µM DEM for 24 h resulted only in a slight non-significant elevation of numbers of worms with predominantly nuclear DAF-16. However, 1 mM DEM significantly increased numbers of worms with predominantly nuclear DAF-16 and significantly decreased those of worms with mostly cytoplasmic localization of DAF-16 (Fig. 4A). Interestingly, the other thiol-modulating compounds, menadione and diamide, also induced a nuclear translocation of DAF-16 – even at 100 µM (Fig. 4B and C). These data indicate that thiol-modulating compounds affect subcellular DAF-16 localization; and by supporting nuclear accumulation, they establish a prerequisite for DAF-16 activity as a transcriptional regulator. On the other hand – and somewhat surprisingly –, these data also imply that DAF-16::GFP nuclear translocation elicited acutely upon exposure to a stressful stimulus not necessarily correlates with a permanently enhanced stress resistance and life span. If anything, strong nuclear accumulation of DAF-16::GFP correlates with impaired stress resistance and shorter life span, such as in the cases of menadione (100 µM) and DEM (1 mM).

Although no nuclear accumulation of the DAF-16 fusion protein was detected in worms exposed to 100 µM DEM for 24 h, Fig. 3A clearly indicates that DAF-16 is required for the life span extending effect of that moderate concentration of DEM. In order to address this discrepancy and to test for a longer term (rather than acute) effect of DEM (100 µM) on DAF-16 activity, we went on to analyze mRNA levels of genes known to be regulated by DAF-16. As SKN-1 also appears to be required for DEM-induced life span extension (Fig. 3B), we similarly analyzed mRNA levels of SKN-1-regulated genes.

### 3.5 Upregulation of DAF-16 and SKN-1 target genes by DEM

Time-dependent changes in mRNA levels of several DAF-16 or SKN-1 target genes were analyzed in worms grown on moderate (100 µM) or high (1 mM) concentrations of DEM for up to 10 days.

**Fig. 5:**
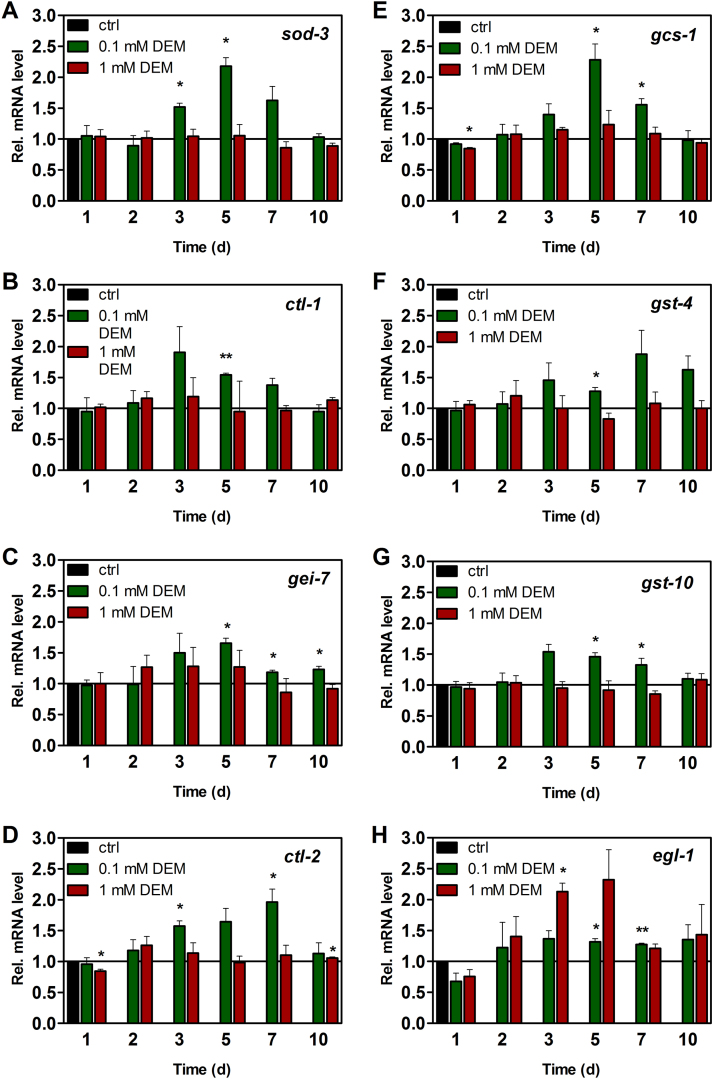
Expression of DAF-16 and SKN-1 target genes after long-term exposure to DEM. Relative mRNA levels of (A-C) predominantly DAF-16-regulated genes, (E-G) predominantly SKN-1-regulated genes, (D) *ctl-2*, a DAF-16 and SKN-1-regulated gene, and (H) *egl-1*, a p53-regulated gene, are depicted. Nematodes were exposed to 100 µM or 1 mM DEM for the given periods of time, collected, and RNA was isolated. The respective mRNA levels were determined by qRT-PCR and normalized over *act-1* mRNA levels as a housekeeper; controls were set to 1. Data are means of 3 independent experiments + SEM (*p<0.05; ^**^p<0.01, Student´s t-test).

We tested for the expression of genes that are known to be regulated by DAF-16: genes involved in antioxidant defense and stress response, *sod-3* (encoding a manganese superoxide dismutase) [27], [28], [29], [30], *ctl-1* and *ctl-2* (encoding cytosolic and peroxisomal catalase, respectively) [28] as well as *gei-7* (aka *icl-1*, encoding an isocitrate lyase) [28] (Fig. 5A–C). Of these, *ctl-2* (Fig. 5D) was also demonstrated to be regulated by SKN-1 [31]. Moreover, we tested for the expression of predominantly SKN-1-regulated genes (*gcs-1*, as well as genes encoding glutathione S-transferases, *gst-4* and *gst-10* [32], [33], [34]; Fig. 5E–G).

Expression of all these DAF-16 or SKN-1-dependent genes was upregulated in worms grown on agar containing 100 µM DEM, whereas no effect on mRNA levels was detected with 1 mM DEM. Time courses slightly varied, but peak mRNA levels were seen after 3–7 days of exposure to 100 µM DEM (Fig. 5A–G). This observation is in line with an adaptive response elicited by DEM, resulting in upregulation of antioxidant genes as well as genes involved in GSH metabolism; for example, the interaction of DEM, a known GST substrate, with GSH is catalyzed by GSTs [21], [35].

As DEM-induced thiol depletion and oxidative stress might result in DNA damage, we also tested for mRNA levels of *egl-1*, a gene regulated by the *C. elegans* p53 orthologue, CEP-1 [36]. Expression of *egl-1* is induced, via CEP-1, by stressful conditions eliciting DNA damage, such as by UVC radiation [37]. It is an activator of apoptotic cell death in *C. elegans* [38]. In contrast to the DAF-16/SKN-1-responsive genes tested, *egl-1* mRNA levels were more strongly upregulated upon exposure to 1 mM DEM (Fig. 5H), consistent with a response running in parallel with the intensity of the stressful stimulus causing damage. Hence, the quality of stress response elicited by 1 mM DEM appears to be fundamentally different from the one elicited at lower DEM concentrations. Whereas low DEM concentrations cause an adaptive (and antioxidant) response, 1 mM DEM elicits damage to an extent that no longer allows for the induction of DAF-16/SKN-1-dependent genes but may rather stimulate signaling processes related to programmed cell death.

### 3.6 Attenuation of glutathione biosynthesis modulates life span and stress resistance of C. elegans

As our data presented above point to a potentially advantageous effect of moderate thiol depletion with regard to antioxidant defense, stress resistance and life span of *C. elegans*, we further aimed at delineating the role of one specific thiol, GSH, in the regulation of stress resistance.

Despite the frequent use of DEM in lowering cellular GSH levels, this is a somewhat unspecific experimental approach as DEM also non-enzymatically forms adducts with other thiols. In fact, we suppose that protein thiols may be primary targets of DEM here, followed only later by attack on GSH (Fig. 1D, E). We therefore specifically targeted GSH by downregulating its *de novo* synthesis through knocking down the rate-limiting enzyme in GSH biosynthesis, γ-glutamylcysteine synthetase (GCS, also called glutamate-cysteine ligase). Using an RNAi approach, we targeted *gcs-1* mRNA, encoding the catalytic subunit (heavy chain) of *C. elegans* GCS-1.

**Fig. 6:**
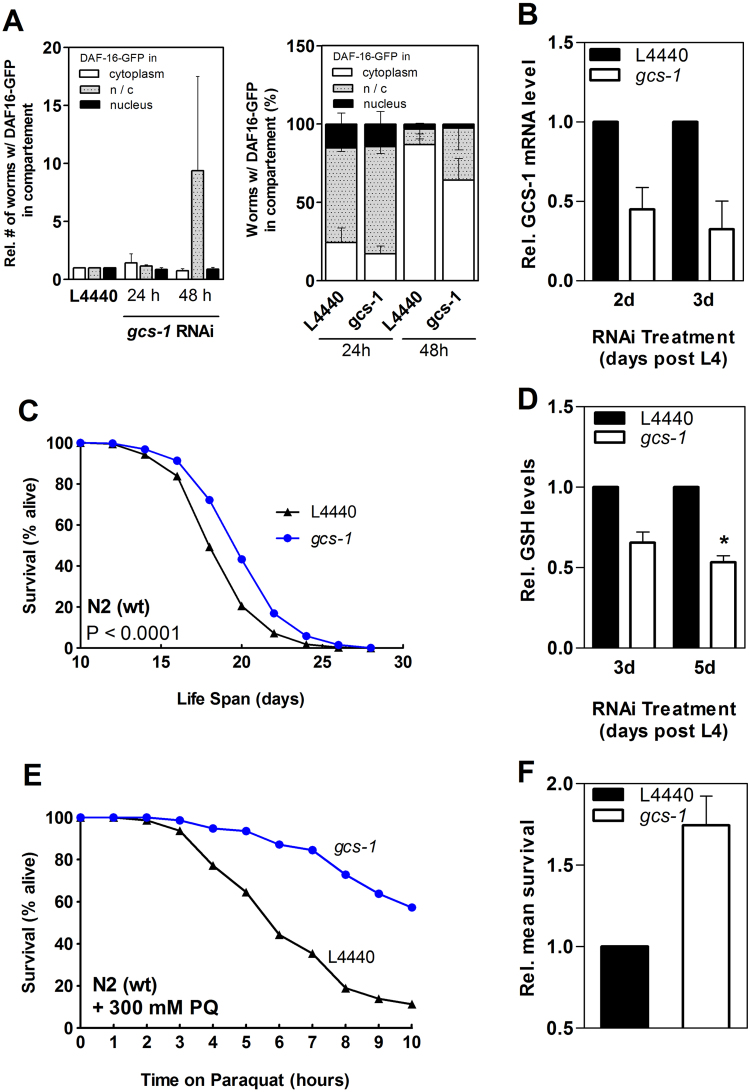
Depletion of γ-glutamylcysteine synthetase starting at young adult stage enhances life span and stress resistance of *C. elegans.* (A) Age-synchronized *C. elegans* TJ356 L1 larvae stably expressing a DAF-16::GFP fusion protein were fed with *gcs-1* RNAi bacteria for 24 and 48 h. DAF-16::GFP localization was analyzed as described in the legend to Fig. 4. Data shown are means+SEM of relative numbers of worms (left) or percentage of worms (right) with DAF-16::GFP in cytoplasm, nucleus or a mixture of both (n/c), as resulting from 3 individual experiments. (B) Relative GCS-1 mRNA levels after 2d or 3d of *gcs-1* RNAi knockdown starting 64 h after synchronization, as determined in three independent experiments using qRT-PCR. Data were normalized to mRNA levels of the housekeeping gene *tba-1* and against the respective controls. Data shown are relative means+SEM. (C) Survival rates of nematodes depleted of GCS-1 through RNAi (blue: *gcs-1*, black: empty vector; p<0.0001, log-rank test). 64 h after synchronization, worms were transferred to NGM agar plates supplemented with 1 mM IPTG and 100 µg/ml ampicillin and spotted with *E. coli* HT115 containing empty vector L4440 or vector containing a *gcs-1* cDNA fragment. Survival at 20 °C was monitored daily until the end of the reproduction period and every second day thereafter. Experiments were conducted in quintuplicates and were performed three independent times (details can be found in Table 5). One representative experiment is shown. (D) Relative glutathione (GSH) levels at day 3 and 5 of adulthood after incubation with *gcs-1* RNAi starting 64 h after synchronization. GSH levels were determined from nematode lysates and were normalized to protein content and the respective control treatments. Relative means+SEM (n=3 independent experiments) are displayed (*p<0.05 vs. control; Student´s t-test). (E) Age-synchronized, 64 h-old wild-type nematodes were incubated for 5 days on agar plates supplemented with 100 μg/ml ampicillin and 1 mM IPTG and spotted with *E. coli* HT115 containing empty vector L4440 (black) or vector containing a *gcs-1* cDNA fragment (blue). N2 wild-type nematodes were transferred to NGM agar plates containing 300 mM paraquat. Survival of nematodes was determined. One representative survival curve is depicted, and relative survivals are presented as means + SEM (3 independent experiments) in (F)**.**

In order to test whether this RNAi approach elicits DEM-like effects on subcellular localization of DAF-16 (see Fig. 4) we first knocked down *gcs-1* mRNA in *C. elegans* stably expressing a DAF-16::GFP fusion protein. DAF-16::GFP localization was not altered after 24 h of feeding RNAi bacteria, whereas a slight increase in the number of worms carrying both nuclear and cytosolic DAF-16::GFP was observed after 48 h (Fig. 6A).

**Fig. 7:**
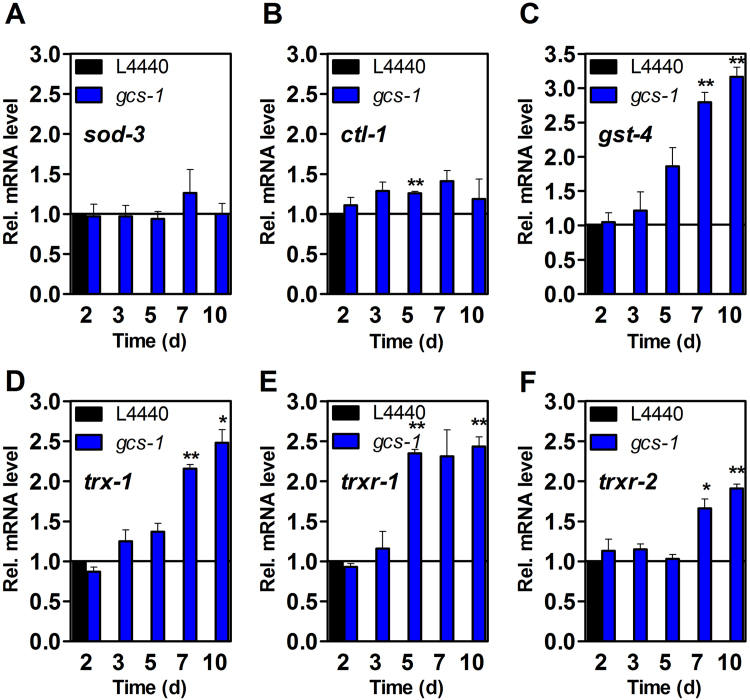
Expression of DAF-16 and SKN-1 target genes and thioredoxin system genes following depletion of γ-glutamylcysteine synthetase (*gcs-1*). Relative mRNA levels of predominantly DAF-16/SKN-1-regulated genes (A–C) as well as thioredoxin (*trx*) and thioredoxin reductase (*trxr*) genes (D–F) are depicted. Age-synchronized, 64 h-old wild-type nematodes were incubated for the given periods of time (up to 10 days) on agar plates supplemented with 100 μg/ml ampicillin and 1 mM IPTG and spotted with *E. coli* HT115 containing empty vector L4440 (black) or vector containing a *gcs-1* cDNA fragment (blue). The respective mRNA levels were determined by qRT-PCR and normalized over *act-1* mRNA levels as a housekeeper; controls were set to 1. Data are means of 3 independent experiments+SEM (*p<0.05; ^**^p<0.01, Student´s t-test).

Young adult wild-type worms with lower *gcs-1* mRNA levels as obtained through RNAi for 3 d and 5 d (Fig. 6B) had significantly lower levels of GSH as compared to nematodes fed with the empty control vector L4440 (Fig. 6D). Lifelong administration of *gcs-1*-specific RNAi bacteria to young adult nematodes (L4) resulted in a mild but significant increase in mean and maximum life span (Fig. 6C, for details, see Table 5), as well as an improved resistance to the oxidative stressor paraquat (Fig. 6E, F). Similar to DEM, depletion of GCS-1 elicited an upregulation of expression of some genes coding for antioxidant proteins: although *sod-3* was not affected, *ctl-1* was slightly and *gst-4* mRNA was strongly upregulated in response to GCS-1/GSH depletion (Fig. 7A–C). GCS-1 depletion appears to have affected the thioredoxin-dependent redox system, as the expression of genes encoding a thioredoxin (*trx-1*, Fig. 7D) as well as two thioredoxin reductases (trxr-1, trxr-2, Fig. 7E–F), was strongly upregulated. The thioredoxin/thioredoxin reductase system contributes to both antioxidant protection and redox regulation of cellular processes [39], and its upregulation may also be interpreted as an adaptive response to GSH depletion. Moreover, GCS-1 depletion resulted in adaptive upregulation of *mtl-1* (data not shown), which encodes metallothionein, a protein that may serve as additional intracellular redox buffer due to its multiple cysteine residues [40].

**Fig. 8:**
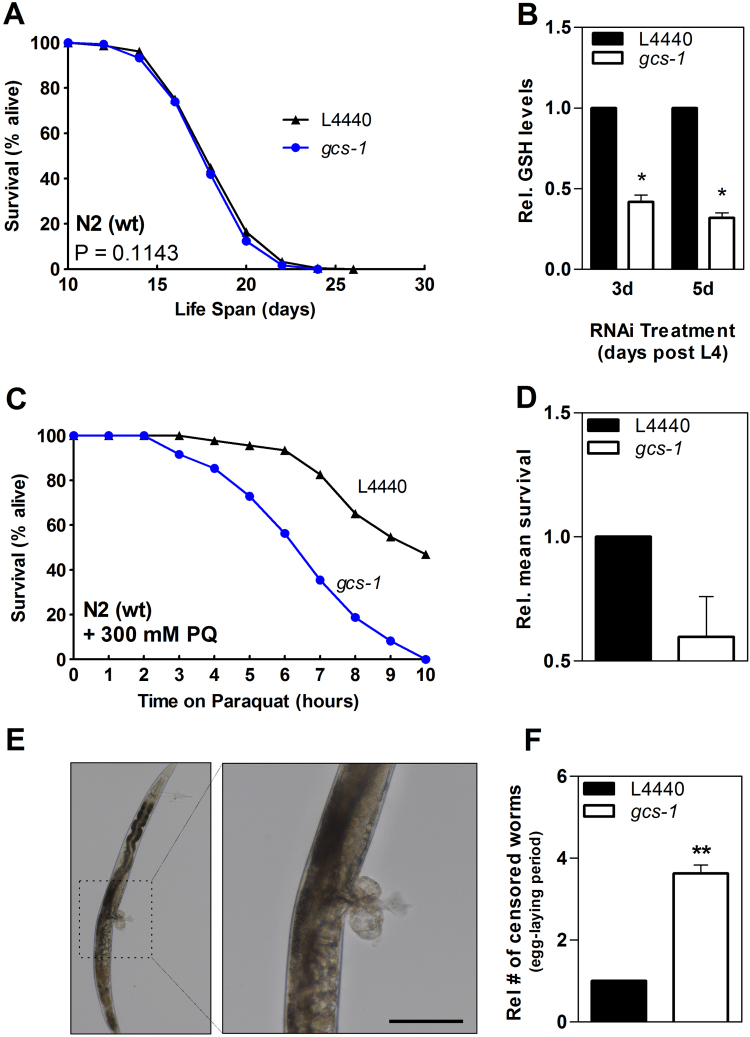
Effects of γ-glutamylcysteine synthetase depletion starting immediately after synchronization on *C. elegans* life span and stress resistance. (A) Survival rates of nematodes depleted of GCS-1 through RNAi (blue: *gcs-1*, black: empty vector); immediately after synchronization, worms were transferred to NGM agar plates supplemented with 1 mM IPTG and 100 µg/ml ampicillin and spotted with *E. coli* HT115 containing empty vector L4440 or vector containing a *gcs-1* cDNA fragment. Survival at 20 °C was monitored daily until the end of the reproduction period and every second day thereafter. Experiments were conducted in quintuplicates and were performed three independent times (details can be found in Table 5). One representative experiment is shown. (B) Relative GSH levels at day 3 and 5 of adulthood (counting from young-adult stage) after incubation with *gcs-1* RNAi starting immediately after synchronization. See legend to Fig. 6D. Data are relative means+SEM (n=3; *p<0.05; Student´s t-test). (C) Immediately after synchronization, wild-type nematodes were transferred to agar plates supplemented with 100 μg/ml ampicillin and 1 mM IPTG and spotted with *E. coli* HT115 containing empty vector L4440 (black) or vector containing a *gcs-1* cDNA fragment (blue). They were grown until young adult stage (64 h) and for 5 d thereafter. N2 wild-type nematodes were then transferred to NGM agar plates containing 300 mM paraquat. Survival of nematodes was determined. One representative survival curve is depicted, and relative mean survivals of 3 independent experiments are presented as means+SEM in (D). (E) Ruptured area of nematode (aged 5 d) depleted of *gcs-1* by RNAi starting immediately after synchronization; right: detail, bar: 100 µm. (F) Relative number of censored worms during egg-laying period in life span analyses with *gcs-1* RNAi or L4440 empty vector starting lifelong incubation immediately after synchronization. Means + SEM from three independent lifespan analyses are displayed (*p<0.05; ^**^p<0.01, Student´s t-test).

The outcome of *gcs-1* knockdown was dramatically different if RNAi started right after synchronization: no effect on lifespan was detected (Fig. 8A), and survival on paraquat was clearly reduced rather than enhanced under these conditions (Fig. 8C, D). Moreover, GSH depletion was more efficient (Fig. 8B). Similar findings on impaired stress resistance of *C. elegans* following *gcs-1* knockdown commencing at L1 stage were previously reported: GCS-deficient worms were more sensitive towards the redox cycler, juglone, and dramatically more susceptible towards arsenite [13].

It should be noted as well that 30–40% of the nematodes in the group with RNAi downregulation of *gcs-1* expression commencing right after synchronization had to be censored due to ruptures at vulvae during their egg-laying period (Fig. 8E, F). In addition, these *gcs-1* RNAi-treated worms appeared thinner than worms of the control group (not shown).

In summary, RNAi-induced depletion of GCS-1 (and therefore GSH) starting at L4 stage enhanced *C. elegans* stress resistance and life span, whereas this beneficial effect was not observed if RNAi commenced immediately after synchronization.

## 4 Discussion

Modulation of GSH levels in *C. elegans* has previously been shown to affect stress resistance and viability. For example, dietary supplementation of worms with a GSH derivative, 5-linolenoyl glutathione, increased lifespan and stress resistance via activation of DAF-16 and up-regulation of sirtuins [41]. An increase in GSH levels was observed in *C. elegans* exposed to moderate concentrations (40–250 µM) of the ROS generator juglone; this was associated with nuclear translocation of the transcription factor DAF-16, but only the lowest concentration of juglone resulted in lifespan extension [22], [42]. In contrast to these previous studies, we observed an increased life span in worms exposed to conditions lowering (rather than elevating) glutathione levels, if their GSH synthesis was knocked down post-L4 stage. We here demonstrate that moderate depletion of GSH – both chemically and through RNAi-induced attenuation of GSH biosynthesis – may enhance *C. elegans* stress resistance and life span.

If slight depletion of GSH enhances life span, then why aren’t basal GSH levels lower in the first place? One potential answer is provided by data depicted in Fig. 8: early stages in *C. elegans* development seem to require adequate GSH availability.

*Role of GSH in development –* Worms with lower maternal and zygotic *gcs-1* activity have recently been shown to arrest in the molt [43]; similarly, cytoplasmic glutathione reductase (GSR-1) activity was shown to be essential for *C. elegans* development, including molting stages [43], [44]. Oxidation of thiols through treatment with 18 mM diamide at the L2 larval stage resulted in arrest during molt as well as in failure of division of vulval equivalence group cells [43], i.e. cells in the vicinity of the very area ruptured in worms undergoing RNAi for *gcs-1* starting prior to L4 (Fig. 8E, F). While these data point to the essentiality of GSH for early nematode development, we here demonstrated that – under standard growth conditions – those *gcs-1(RNAi)* worms surviving beyond L4 stage under conditions of GSH depletion do not differ in life expectancy from control worms (Fig. 8A). However, these worms were much more sensitive to a stressful environment and less capable of resisting exposure to the redox cycler, paraquat (Fig. 8C, D). In contrast, depletion of GSH at a later point in life, commencing at L4, rendered worms more resistant towards oxidative stress (Fig. 6E, F), resulting in an extended life span (Fig. 6C).

One important contribution of GSH during development until L4 was demonstrated to be the reduction of cuticle proteins, in concert with the thioredoxin-dependent redox system [43], during various molting steps. It appears that GSH depletion prior to L4 is thus perceived by *C. elegans* as harsh stress, inducing survival mode rather than adaptation, hence not contributing to an enhanced stress resistance even after reaching young adult stage (see Fig. 8C, D). Yet after reaching L4, that same RNAi treatment – as well as exposure to moderate concentrations of DEM – is a stimulus initiating an adaptive response.

*Molecular basis of the observed hormetic effect of DEM* – DEM, through depletion of thiols, contributes to a net increase in the steady-state levels of reactive oxygen species (as demonstrated by oxidation of dihydrodichlorofluorescein to form fluorescent DCF; data not shown). Thus, the increases in life span and stress resistance induced by low doses of DEM as described above would be in line with previous reports on hormetic effects of oxidative stress. The adaptive response elicited by mild sub-lethal oxidative stress may result in an improved stress resistance and elevated life expectancy of *C. elegans*, as long as ROS levels do not exceed the protective capacity of the intrinsic antioxidant systems [19], [22], [45], [46]. In cells under conditions of mild oxidative stress, thiol groups in thioredoxin and other proteins including redox-sensitive enzymes and transcription factors are usually oxidized first; this results in alterations of their activity and altered signal transduction [47]. A further increase in ROS levels causes severe oxidative stress and oxidation of a substantial fraction of GSH [47].

Other thiol-reactive compounds, including the synthetic vitamin K analog menadione (2-methyl-1,4-naphthoquinone, see also Fig. 1B) and the phytochemical plumbagin (5-hydroxy-2-methyl-1,4-naphthoquinone) have recently been shown to affect the life span of *C. elegans*: both compounds were toxic at doses ≥100 µM, whereas plumbagin but not menadione extended the mean life span of the worms when applied at lower doses ranging from 25 to 60 µM [19]. Both menadione and plumbagin are redox cyclers and ROS generators and are also capable of depleting cells of GSH and other thiols through alkylation [18], [48], [49]. It has been proposed that modulation of cellular thiols, rather than ROS generation, may play a major role in the biological actions of plumbagin, including signaling effects [49].

We found that the life span-extending effect of DEM in *C. elegans* is mediated by DAF-16 and SKN-1 (Fig. 3); these transcription factors are known to regulate stress and antioxidant response in *C. elegans* [32], [50]. It has previously been shown that GSH can modulate the DAF-16 pathway, likely through the sirtuin, SIR-2.1 [41]. We found the expression of prominent target genes of both transcription factors significantly upregulated in worms cultured in the presence of 100 µM DEM, including antioxidant target genes regulated by DAF-16, *sod-3*, *ctl-1 and ctl-2* [27], [28], [29], [32] as well as three predominantly SKN-1-dependent glutathione-related genes, *gcs-1* (the gene coding for γ-glutamylcysteine syntethase-1), and two glutathione-S-transferase genes, *gst-4* and *gst-10* [30], [32], [33], [34].

Despite the above hints on how a toxicant such as DEM can elicit beneficial effects at low to moderate concentrations, the exact molecular basis of the DEM-induced adaptive response requires further elucidation. It appears that although DEM at 1 mM triggers nuclear accumulation of DAF-16 (Fig. 4A), it does not cause prominent stimulation of DAF-16 target genes (Fig. 5). At this point, two explanatory lines of speculation are as follows: nuclear glutathione may be affected in a way prohibiting the activity or regulation of certain redox-sensitive transcription factors, e.g. through alteration of glutathionylation patterns [51], [52]. Alternatively, DEM may, by alkylating susceptible thiols, directly interfere with transcription factor activity and/or subcellular localization; for example, the nuclear export machinery in mammalian nuclei was demonstrated to be impaired by DEM [53], suggesting that it can “freeze” factors inside nuclei, even in their inactive forms.

## Conflict of interest

The authors declare no conflict of interest.

## Author contributions

NU and LOK conceived study. NU and DT performed experiments for DEM. KK did life span analysis for menadione and diamide; KK and KE performed GSH measurements. FH, DT and NU performed *gcs-1* RNAi experiments and collected samples for HPLC. VS performed localization experiments. MR provided essential reagents. NU, HS and LOK wrote the manuscript.

## Figures and Tables

**Table 1:** Primer pairs used for qPCR analyses.

**Gene name**	**Gene ID**	**Forward primer (5′→3′)**	**Reverse primer (5′→3′)**
*act‐1*	NM_073418	ATCAAGATCATCGCCCCACC	GCCGGACTCGTCGTATTCTT
*ctl‐1*	NM_064578	TCGTTCATGCCAAGGGAGC	GATCCCGATTCTCCAGCGAC
*ctl‐2*	NM_001027302	GAAGGTGTTGGATACCGGGG	GGATGAGTGCCTTGACACGA
*egl‐1*	NM_074174	AGATCAGCAGCATCGGCTAC	CATGGGCCGAGTAGGACATC
*gcs‐1*	NM_063526	TGTGAACGTCGATGAAGCCA	TCCACGGAAGATTGGTGTGG
*gei‐7*	NM_001026196	CTGCCATCTCCGTGGTATCC	ACCCATGTTCCATCGTGTCC
*gst‐4*	NM_069447	GCCCGTGATGATTTCTTGGC	GCCCAAGTCAATGAGTCTCCA
*gst‐10*	NM_071300	ATCCAACGAGCAAGAGGCAA	ACTTCACTAGAGCCTCCGGG
*sod‐3*	NM_078363	CCACCTGTGCAAACCAGGAT	TGCAAGTAGTAGGCGTGCTC
*tba‐1*[54]	NM_001264284	TCAACACTGCCATCGCCGCC	TCCAAGCGAGACCAGGCTTCAG
*trx‐1*	NM_001026714	ATGTCGATGAAGCGGAAGAT	TTTGACGCAGTTCGTCCTC
*trxr‐1*	NM_001307310	CAAGCGACAGCCGAGACAA	ACTCGCTGCTACTCCCATAGA
*trxr‐2*	NM_066570	CTCAACCGTCGGGTTAACTG	TCGATCGAATCTTCTCCATGT

**Table 2:** Statistics for DEM, menadione and diamide lifespan analyses.

Exp. No.	Strain, treatment	Effect on life span	*P* (vs. Ctrl)^**a**^	Mean life span (days±SEM)^**b**^	Mean life span (%)	Max life span (days±SEM)^**b,c**^	Max life span (%)	No. of uncensored worms		Total No.
**Exposure to DEM**										
**1**	N2/DMSO			22.53±0.2	100	25.6±0.4	100		303	400
	N2/100 µM DEM	**↑**	^****^	23.76±0.1	105.43	26.0±0.0	101.56		297	400
**2**	N2/DMSO			22.67±0.1	100	26.0±0.0	100		288	400
	N2/1 µM DEM	=	n.s.	22.84±0.1	100.75	26.0±0.0	100.00		289	400
	N2/10 µM DEM	=	n.s.	22.99±0.1	101.43	26.0±0.0	100.00		305	400
	N2/100 µM DEM	**↑**	^*^	23.42±0.3	103.32	26.8±0.5	103.08		305	400
	N2/1 mM DEM	**↓**	^****^	20.82±0.3	91.83	23.2±0.5	89.23		293	400
**3** (see Fig. 1C)	N2/DMSO			21.62±0.1	100	24.0±0.0	100		244	400
	N2/1 µM DEM	=	n.s.	21.82±0.2	100.90	24.4±0.4	101.67		202	400
	N2/10 µM DEM	=	n.s.	22.07±0.2	102.07	24.8±0.5	103.33		210	400
	N2/100 µM DEM	**↑**	^**^	22.74±0.1	105.18	26.0±0.0	108.33		220	400
	N2/1 mM DEM	**↓**	^***^	20.51±0.1	94.87	23.6±0.4	98.33		250	400
**Exposure to DEM on heat-inactivated (HIT) bacteria**										
**1**	N2/DMSO			22.95±0.1	100	24.0±0.0	100		248	402
	N2/1 µM DEM	**↑**	^****^	24.87±0.2	108.34	27.2±0.5	113.33		174	405
	N2/10 µM DEM	**↑**	^****^	24.45±0.2	106.53	28.0±0.0	116.67		177	404
	N2/100 µM DEM	=	n.s.	23.18±0.2	100.98	25.6±0.4	106.67		165	402
	N2/1 mM DEM	**↓**	^****^	18.70±0.3	81.47	20.4±0.4	85.00		260	403
**Exposure to Menadione (MQ)**										
**1**	N2/DMSO			20.83±0.4	100	22.4±0.4	100		216	401
	N2/100 µM MQ	**↓**	^****^	17.90±0.4	85.94	19.6±0.4	87.50		259	400
**2** (see Fig. 1B)	N2/DMSO			20.11±0.4	100	21.6±0.7	100		206	400
	N2/100 µM MQ	**↓**	^*^	19.47±0.1	96.83	21.6±0.4	100.00		263	400
**Exposure to Diamide (Dia)**										
**1** (see Fig. 1A)	N2/DMSO			18.68±0.5	100	20.4±0.4	100		200	425
	N2/100 µM Dia	=	n.s.	18.97±0.3	101.52	20.0±0.6	98.04		166	425
**2**	N2/DMSO			19.79±0.3	100	21.6±0.4	100		211	400
	N2/100 µM Dia	=	n.s.	19.58±0.2	98.93	20.8±0.5	96.30		227	400

**^a^**Control: N2/DMSO; * P<0.05; ** P<0.01; *** P<0.001; **** P<0.0001; n.s., not significant; **^b^**5 technical replicates; **^c^**75% quantile.

**Table 3:** Statistics for stress resistance analysis.

Exp. No.	Strain, treatment	Effect on survival		*P* (vs. Ctrl)^a^	Mean Survival (days±SEM)^b^	Mean survival (%)	Max survival (days±SEM)^b,c^	Max survival (%)	No. of uncen-sored worms	Total No.
**Stress resistance against paraquat**										
**1** (see Fig. 2)	N2/ DMSO				4.44±0.2	100	5.3±0.3	100	265	300
	N2/1 µM DEM		=	n.s.	4.29±0.2	96.49	5.0±0.0	93.75	143	146
	N2/10 µM DEM		**↑**	^****^	4.83±0.1	108.60	5.7±0.3	106.25	295	300
	N2/100 µM DEM		=	n.s.	4.27±0.1	96.00	5.0±0.0	93.75	289	300
	N2/1 mM DEM		**↓**	^****^	3.24±0.2	72.96	4.0±0.0	75.00	290	300
**2**	N2/DMSO				4.41±0.1	100	5.0±0.0	100	281	300
	N2/1 µM DEM		=	n.s.	4.27±0.1	96.83	5.0±0.0	100.00	287	300
	N2/10 µM DEM		**↑**	^*^	4.64±0.4	105.24	5.7±0.3	113.33	281	300
	N2/100 µM DEM		=	n.s.	4.55±0.1	103.24	5.3±0.3	106.67	285	300
	N2/1 mM DEM		**↓**	^****^	3.89±0.1	88.17	4.7±0.3	93.33	234	300

**^a^** Control: N2/DMSO, heat-inactivated OP50; * P<0.05; **** P<0.0001; n.s., not significant; ^**b**^ 3 technical replicates; **^c^** 75% quantile

**Table 4:** Statistics for *daf-16* and *skn-1* knockout mutant lifespan analyses.

Exp. No.	Strain, treatment	Effect on life span	*P* (vs. Ctrl)**^a^**	Mean life span (days±SEM)^**b**^	Mean life span (%)	Max life span (days±SEM)^**b,c**^	Max life span (%)	No of uncen-sored Worms		Total No.
***daf‐16*****(*****mu86*****)**										
**1**	*daf‐16*/DMSO			21.38±0.1	100	24.0±0.0	100		279	400
	*daf‐16*/100 µM DEM	=	n.s.	21.72±0.2	101.56	24.8±0.5	103.33		293	400
**2**	*daf‐16*/DMSO			21.11±0.2	100	23.6±0.4	100		265	400
	*daf‐16*/100 µM DEM	=	n.s.	21.30±0.2	100.90	24.0±0.0	101.70		278	400
**3** (see Fig. 3A)	*daf‐16*/DMSO			19.50±0.1	100	22.4±0.4	100		321	400
	*daf‐16*/100 µM DEM	=	n.s.	19.50±0.2	99.99	22.8±0.5	101.79		332	400
	*daf‐16*/1 mM DEM	**↓**	^**^	18.46±0.1	94.67	21.6±0.4	96.43		305	400
**4**	*daf‐16*/DMSO			19.80±0.2	100	21.2±0.5	100		252	400
	*daf‐16*/100 µM DEM	**↓**	^*^	19.36±0.1	97.81	20.4±0.4	96.23		324	400
	*daf‐16*/1 mM DEM	**↓**	^****^	18.52±0.1	93.55	20.0±0.0	94.34		322	400
***skn‐1*****(*****zu135*****)**										
**1**	*skn‐1*/DMSO			20.88±0.2	100	22.8±0.5	100		240	276
	*skn‐1*/100 µM DEM	=	n.s.	21.35±0.2	102.26	22.8±0.5	100.00		258	284
	*skn‐1*/1 mM DEM	**↓**	^**^	19.93±0.1	95.44	22.0±0.0	96.49		255	292
**2** (see Fig. 3B)	*skn‐1*/DMSO			19.91±0.3	100	21.6±0.4	100		142	156
	*skn‐1*/100 µM DEM	=	n.s.	19.99±0.1	100.42	22.2±0.7	102.78		142	150
	*skn‐1*/1 mM DEM	**↓**	^****^	18.34±0.4	92.15	20.4±0.4	94.44		132	144

**^a^** Control: *daf-16*/DMSO or *skn-1*/DMSO; * P<0.05; ** P<0.01; **** P<0.0001; n.s., not significant; ^**b**^ 5 technical replicates; **^c^** 75% quantile

**Table 5:** Statistics for *gcs-1* RNAi lifespan analysis.

Exp. No.	Strain, treatment	Effect on life span	*P* (vs. Ctrl)**^a^**	Mean life span (days±SEM)^**b**^	Mean life span (%)	Max life span (days±SEM)^**b,c**^	Max life span (%)	No of uncen-sored worms		Total No.
***gcs‐1*****RNA interference (L4)**										
**1**	N2/L4440 (L4)			21.24±0.1	100	23.2±0.5	100		334	402
	N2/*gcs‐1* (L4)	**↑**	^****^	22.29±0.2	104.94	24.8±0.5	106.90		342	398
**2**	N2/L4440 (L4)			20.21±0.2	100	22.4±0.4	100		348	400
	N2/*gcs‐1* (L4)	**↑**	^**^	21.09±0.1	104.38	23.2±0.5	103.57		334	400
**3** (see Fig. 6C)	N2/L4440 (L4)			19.15±0.2	100	20.4±0.4	100		334	400
	N2/*gcs‐1* (L4)	**↑**	^****^	20.55±0.1	107.34	22.0±0.0	107.84		344	400
***gcs‐1*****RNA interference (L1)**										
**1**	N2/L4440 (L1)			17.57±0.1	100	18.4±0.4	100		414	500
	N2/*gcs‐1* (L1)	=	n.s.	17.76±0.1	101.09	18.8±0.4	102.17		336	600
**2**	N2/L4440 (L1)			17.66±0.1	100	19.6±0.4	100		410	500
	N2/*gcs‐1* (L1)	**↑**	^**^	18.21±0.1	103.12	20.0±0.0	102.04		290	500
**3** (see Fig. 8A)	N2/L4440 (L1)			18.71±0.1	100	20.0±0.0	100		417	500
	N2/*gcs‐1* (L1)	=	n.s	18.47±0.2	98.73	20.0±0.0	100		295	500

**^a^** Control: N2/L4440; ** P<0.01; **** P<0.0001; n.s., not significant; ^**b**^ 5 technical replicates; **^c^** 75% quantile.
